# Electroconvulsive Therapy for the Treatment of Somatic Delusions

**DOI:** 10.7759/cureus.54577

**Published:** 2024-02-20

**Authors:** Brittnie Phan, Chong Yang

**Affiliations:** 1 Psychiatry, Touro University College of Osteopathic Medicine, Vallejo, USA; 2 Psychiatry, Department of State Hospitals-Napa, Napa, USA

**Keywords:** efficacy of ect, delusions, somatic delusions, schizoaffective disorder, electroconvulsive therapy (ect)

## Abstract

Somatic delusions occur in various psychiatric disorders and are associated with higher mortality and lower quality of life. In this case report, we present a 68-year-old man with the diagnosis of schizoaffective disorder, bipolar type with associated somatic delusions, and auditory hallucinations. His somatic delusions were alleviated by the 20^th^ ECT treatment with additional clinical improvements in his speech, thought processes, and judgment. This case report supports the utilization of ECT for patients with schizoaffective disorder and somatic delusions.

## Introduction

Somatic delusions are false and fixed beliefs about health and organ function that occur in various psychiatric disorders such as schizophrenia, major depressive disorder, and bipolar disorder. These delusions can be very challenging to treat and are associated with increased violence, lower quality of life, and greater risk for suicide [1,2]. In a large sample of patients with their first episode of psychosis, somatic delusions were prominent in 18% [1]. Historically, somatic delusions were considered to be a treatment-resistant disorder; However, there is evidence that pimozide has been effective in the treatment of somatic delusions [3]. Other studies supported the use of risperidone and olanzapine, but the efficacy of these medications varied according to the presence or absence of specific symptoms such as cognitive defect and depression. Finally, clozapine is considered to be the most effective, but its use is limited due to its potential side effects [3]. 

If a patient cannot tolerate pharmacologic therapies, such as clozapine due to its side effects, electroconvulsive therapy (ECT) may be an option. One study showed that clozapine and ECT share similar mechanisms in the treatment of psychiatric disorders [4]. Both treatment modalities elicit alter electroencephalogram (EEG) activity, increase neuroplasticity and elevate brain levels of neurotrophic factors, affect glutamate and gamma-aminobutyric acid’s relationship, and reduce inflammation through neuron-glia interactions [4]. Somatic delusions are associated with reduced regional cerebral blood flow (rCBF), and ECT’s mechanism in altering the rCBF may be responsible for the alleviation of somatic delusions [5]. Several case studies have reported the successful treatment of somatic delusions with ECT. Ranjan reported the resolution of multiple somatic delusions with ECT in a patient with late-onset persistent delusional disorder [6]. Similarly, Hayashi reviewed the efficacy of ECT in a case report of a patient diagnosed with schizoaffective disorder with severe and persistent somatic delusions that were resistant to several antipsychotics and antidepressants [5]. In the following case report, we present a patient diagnosed with schizoaffective disorder with somatic delusions who was treated with ECT.

## Case presentation

The patient was a 68-year-old male diagnosed with schizoaffective disorder, bipolar type. He had a history of psychosis which included delusions, auditory hallucinations, and disorganized thinking. He also had a history of mania which included grandiose beliefs, increased energy, decreased need for sleep, pressured speech, and increased goal-directed activity. He endorsed the somatic delusion that voices in his mind were controlling the blades that were going over his feet, causing pain in his feet. His psychotic and mood symptoms have not responded to the following psychotropic medication trials: valproic acid (up to 2000 mg/day), risperidone (up to 6 mg/day), and haloperidol (up to 15 mg/day). Clozapine (up to 100 mg/day) was taken for ten years but discontinued due to the development of severe neutropenia on the medication. Prior to his ECT treatments, he remained adherent to olanzapine 40 mg/day.

Due to his partial response to olanzapine and his history of neutropenia on clozapine, ECT was considered. Treatment parameters included bitemporal stimulus electrode placement, 1 milligram/kilogram (mg/kg) of methohexital as the anesthetic, and 1 mg/kg of succinylcholine as the muscle relaxant. Per hospital policy, a therapeutic seizure was defined as a seizure of 20 seconds or longer as evident by EEG and the psychiatrist has a maximum of three attempts (delivery of the electrical stimulus) to obtain a therapeutic seizure for any given ECT treatment session. The seizure threshold was established at 202 mC at the second ECT treatment session. The index course of ECT treatment consisted of three ECT treatments per week. The results of his 20 treatments are summarized in Table 1. By the 20th ECT treatment, the patient reported a resolution of his foot pain, and his ECT treatments were tapered down to twice per week. 

**Table 1:** Multiple energy levels were presented in treatments when multiple attempts occurred to reach the therapeutic seizure level

Treatment	Energy Charge (mC)	EEG seizure (sec)
1	50.4; 101; 151	N/A
2	202; 252	98
3	252; 302; 353	119
4	353	96
5	353	103
6	353; 403	69
7	403; 403; 454	N/A
8	504; 504; 504	35
9	504	65
10	504; 504	N/A
11	504	31
12	504	46
13	504; 504; 504	N/A
14	504	48
15	504	99
16	504	32
17	504	130
18	504	64
19	504; 504; 504	NA
20	504	128

## Discussion

At times, the ECT psychiatrist was unable to obtain a therapeutic seizure. Despite a titration of the stimulus dose, we were unable to obtain a therapeutic seizure at treatment #7. Similarly, we were unable to obtain a therapeutic seizure for treatments #10, 13, and 19 despite the maximum stimulus dose. The patient was only taking olanzapine but not taking any mood stabilizers or benzodiazepines that would alter the seizure threshold. Caffeine dosages starting at 60 mg and up to 600 mg were provided to elicit a therapeutic seizure, since studies have shown that caffeine increases seizure susceptibility and has decreased the antiepileptic potency of some drugs, such as topiramate [7]. 

On admission, the patient had disorganized and loosely connected thoughts and the admitting psychiatrist stated he was unable to answer basic questions without tangents and repeated redirection. After his ECT treatments, his speech became coherent, his thoughts became linear and goal-directed, and his judgment improved. Furthermore, he demonstrated flexibility in thinking and understood that ECT helped him achieve mental stability. Although his auditory hallucinations persist, he endorsed the successful resolution of his somatic delusions after the 20th treatment of ECT. This indicates the mechanism in which ECT improves overall psychosis and somatic delusions. Several case reports show that somatic delusions typically resolve after a course of 6-10 sessions of bilateral ECT treatments [6].

The patient presented with confusion and memory problems after ECT treatments. In the index course of treatments, patients reported that the most negative aspect of ECT was memory impairment [8]. However, in the continuous/maintenance phase of ECT (C/M-ECT), there were no objective measures of cognitive impairment due to the longer time interval between treatments [8]. Most studies have shown that long-term C/M-ECT has been associated with a slight increase in cognitive function with improvement on the mini mental state examination and, evidence indicates that abrupt early cessation of ECT increases the chance of recurrence [8,9]. Therefore, our patient may benefit from continuous/maintenance ECT, especially since he previously used clozapine for ten years and clozapine and ECT share similar mechanisms in resolving psychosis [9,10]. In a randomized controlled study that evaluated C-ECT in patients with schizophrenia, combined treatment was considerably more effective than drug therapy or C-ECT administered separately [10]. Therefore, patients who were resistant to pharmacotherapy previously often responded to C-ECT, which implies a rekindling effect on pharmacological treatment. The literature shows that current pharmacological treatments seem to be more efficacious at treating hallucinations rather than delusions, so the combination of maintenance ECT and medications may help alleviate both the delusions and hallucinations in this patient [1]. We still need more studies including patients that utilize the combination of ECT and pharmacologic treatment and larger sample sizes to determine if C-ECT treatment is an effective option for this patient population. 

## Conclusions

Patients with somatic delusions experiencing other psychotic symptoms such as auditory hallucinations may benefit from ECT. The literature on the efficacy of ECT for patients with somatic delusions is supported by several case reports. Our case report supports the utilization of ECT for patients diagnosed with schizoaffective disorder with somatic delusions. However, we need additional studies to support our findings and longitudinal studies to understand the long-term outcomes of these patients. 
